# Cytotoxic diagnostic assays are effective in drug-induced maculopapular exanthemas

**DOI:** 10.1186/2045-7022-5-S1-O18

**Published:** 2015-03-11

**Authors:** Grzegorz Porebski, Magdalena Bosak

**Affiliations:** 1 Jagiellonian University, Dep. of Clinical and Environmental Allergology, Krakow; Poland; 2 Jagiellonian University, Department of Neurology, Krakow, Poland

## Background

Cytotoxic mechanisms are involved in most forms of drug-induced skin diseases, but till now only a small number of studies has been addressed the question whether in vitro detection of cytotoxic drug-specific response could be helpful in drug hypersensitivity diagnosis. The aim of this study was to assess and compare different cytotoxic tests as an alternative nonradioactive approach which may be more appropriate for routine testing and may provide in addition more information about the pathophysiology of the reaction than proliferation-based assays, like the lymphocyte transformation test (LTT).

## Method

Number of granzyme B secreting cells by ELISpot (GrB-ELISpot), perforin concentrations in the supernatants (Per-ELISA) and intracellular expression of granulysin in NKp46+ cells by means of flow cytometry (Grl-NK) were evaluated in drug-stimulated cultures of peripheral blood mononuclear cells from 34 patients in remission state, suspected of antiepileptic drug-induced maculopapular exanthema and 24 drug-exposed healthy controls. Lymphocyte transformation tests with corresponding drugs were performed in all investigated individuals.

## Results

In 18/36 patients tested drugs elicited granzyme B release in ELISpot. Staining for granulysin was positive in 15/36 patients, whereas increased secretion of perforin was detected in 3/36 patients. 11/36 patients showed positive proliferative response to the drug, measured in lymphocyte transformation test. None of the 24 drug-exposed healthy donors reacted to the tested drugs in perforin assay and LTT, however one reacted in GrB-ELISpot and another one in granulysin assay. Obtained data correspond to the following sensitivities and specificities, respectively: GrB-ELISpot: 53% and 96%; Grl-NK: 44% and 96%; Per-ELISA: 9% and 100%; LTT: 32% and 100%.

## Conclusion

In vitro cytotoxic assays of drug-specific GrB-ELISpot and granulysin production in NK cells, but not Per-ELISA offer potential for use as effective diagnostic tests and advantages over the LTT, including a shorter assay time and a greater sensitivity. As a significant percentage of included patients suspected of drug-induced skin reaction did not show any positive response in any test, we can assume that causal relationship between drug exposure and common exanthemas is often overestimated.

